# 

**Published:** 1857-11

**Authors:** 


### CONTENTS.

### ORIGINAL COMMUNICATIONS.

PAGE.
ARTICLE I.—A Thesis on Vesico-Vaginal-Fistula. Presented to the Faculty of the Atlanta Medical College for the Degree of Doctor of Medicine. By CAMILLUS HILLIARD, of Montgomery, Ala., .......... 129

### SELECTIONS.

The St. Louis Medical Society, .......... 137
Mechanism of Nervous Action, .......... 150
Professional Secrecy, .......... 156
Lecture on Cancer Cures and Cancer Curers, .......... 161
Asiatic Cholera and Cholera Morbus, .......... 170
Andrews on the Physiology of the Three Registers of the Human Voice, .......... 177

### EDITORIAL AND MISCELLANEOUS.

Editorial Responsibility, .......... 184
The Medical Regulator Again, .......... 185
Death of Charlotte Bronte, .......... 188
Cold Applications in Pneumonia and Pleurisy, .......... 191
Treatment of the Asphyxia of New-born Children, .......... 192
The Plantago Major in Spider Bite, .......... 192
Subscriptions Received, .......... 192

